# Application and Progress of Chemometrics in Voltammetric Biosensing

**DOI:** 10.3390/bios12070494

**Published:** 2022-07-07

**Authors:** Jingjing Liu, Yifei Xu, Shikun Liu, Shixin Yu, Zhirun Yu, Sze Shin Low

**Affiliations:** 1 College of Automation Engineering, Northeast Electric Power University, Jilin 132012, China; 2202000603@neepu.edu.cn (Y.X.); 2202000530@neepu.edu.cn (S.L.); 2202100652@neepu.edu.cn (S.Y.); 2 College of Law, The Australian National University, Canberra 2600, Australia; yuzhirun@126.com; 3 Research Centre of Life Science and HealthCare, China Beacons Institute, University of Nottingham Ningbo China, 199 Taikang East Road, Ningbo 315100, China

**Keywords:** chemometrics, biosensing, voltammetric methods, smartphones, point-of-care testing

## Abstract

The voltammetric electrochemical sensing method combined with biosensors and multi-sensor systems can quickly, accurately, and reliably analyze the concentration of the main analyte and the overall characteristics of complex samples. Simultaneously, the high-dimensional voltammogram contains the rich electrochemical features of the detected substances. Chemometric methods are important tools for mining valuable information from voltammetric data. Chemometrics can aid voltammetric biosensor calibration and multi-element detection in complex matrix conditions. This review introduces the voltammetric analysis techniques commonly used in the research of voltammetric biosensor and electronic tongues. Then, the research on optimizing voltammetric biosensor results using classical chemometrics is summarized. At the same time, the incorporation of machine learning and deep learning has brought new opportunities to further improve the detection performance of biosensors in complex samples. Finally, smartphones connected with miniaturized voltammetric biosensors and chemometric methods provide a high-quality portable analysis platform that shows great potential in point-of-care testing.

## 1. Introduction

The field of biosensing is in a phase of rapid development, and biosensing assisted by chemometric methods has been gradually introduced to various areas of research. The most common use of voltammetric biosensing is in the the detection of target analytes [1]. The International Union of Pure and Applied Chemistry (IUPAC) defines a biosensor as “an autonomous analytical device capable to performing quantitative or semi-quantitative analyzes using a recognition element (biological in case of biosensor) in direct contact with a transduction element” [2]. Compared with traditional chemical sensors, biosensors have higher selectivity and sensitivity due to the participation of biological elements (enzymes, antibodies, aptamers, molecularly imprinted polymers and DNA, etc.). It provides high resolution and specific response to one or a class of target analytes under certain conditions [3,4,5,6,7,8]. Inspired by the multi-dimensional recognition systems of organisms, voltammetric sensors with low selectivity or specificity are widely used in the detection of biomolecules [9,10,11,12]. They can characterize multicomponent liquid samples holistically by their own cross-sensitivity or be integrated into a sensing system to form a voltammetric detection system, enabling the detection of primary analytes in the presence of interfering substances and the detection of multiple components [13,14].

Voltammetry, which has good sensitivity, detection speed, reliability and accuracy, is one of the most widely used electrochemical techniques in biosensing [15,16,17,18]. It is not only an effective way to investigate the reaction mechanism of a target analyte from an electrochemical point of view, but also can quantify sample parameters using different voltammetric techniques (e.g., cyclic voltammetry, differential pulse voltammetry, square wave pulse voltammetry and anodic dissolution square wave voltammetry). The response current and excitation potential formed during the electroanalysis of a solution forms a voltammogram with high-dimensional characteristics. The inherent richness of voltammetry to generate analytical signals, and the need for researchers to resolve valid information from voltammograms, have promoted the use of chemometrics in voltammetric biosensing. With the combination of chemometrics, biosensors can achieve lower detection limits and better specificity for analytes [19,20]. Meanwhile, chemometric tools can help in mining more meaningful results from the rich information collected by a bioelectronic tongue [21]. It is worth mentioning that machine learning methods and deep learning methods have gradually become new driving forces that continue to promote the development of chemometrics in biosensing [22]. In addition, advances in size reduction, cost lowering and biosensor sensitivity has led to the development of promising applications in point-of-care testing (POCT) [23]. The popularity of smartphones further provides a platform for the use of electrochemical detection devices incorporating chemometric methods in POCT.

The purpose of this review is to show the potential of chemometric methods in biosensing research using voltammetry sensors through an introduction of classical chemometric methods as a tool to solve the limiting problems in biosensing analysis using voltammetry. At the same time, this review stimulates researchers’ interest in using deep learning methods for voltammetric data analysis. Finally, it is envisioned that this review will encourage researchers to combine the advantages of voltammetric analysis and chemometrics in the field of biosensing to develop more smartphone-mediated electrochemical platforms with on-site decision-making capabilities.

## 2. Voltammetric Analysis Techniques Commonly Used in Biosensing

Voltammetry is generally used in standard three-electrode system (working electrode, counter electrode and reference electrode). It applies a certain form of potential to cause the oxidation and reduction reaction of electroactive substances on a working electrode, then samples the response current within the time range [24]. Cyclic voltammetry (CV) [25], differential pulse voltammetry (DPV) [26] and square wave voltammetry (SWV) [27] are common analytical techniques. The selection of a suitable voltammetric technique is helpful for the study of biosensing mechanisms, improving the sensitivity and selectivity of biosensors. A variety of voltammetric methods have been widely used in biosensing in combination with highly sensitive, rapidly responsive electrochemical biosensors. Table 1 lists the biosensing applications using the three voltammetric methods mentioned above.

**Figure 1:**
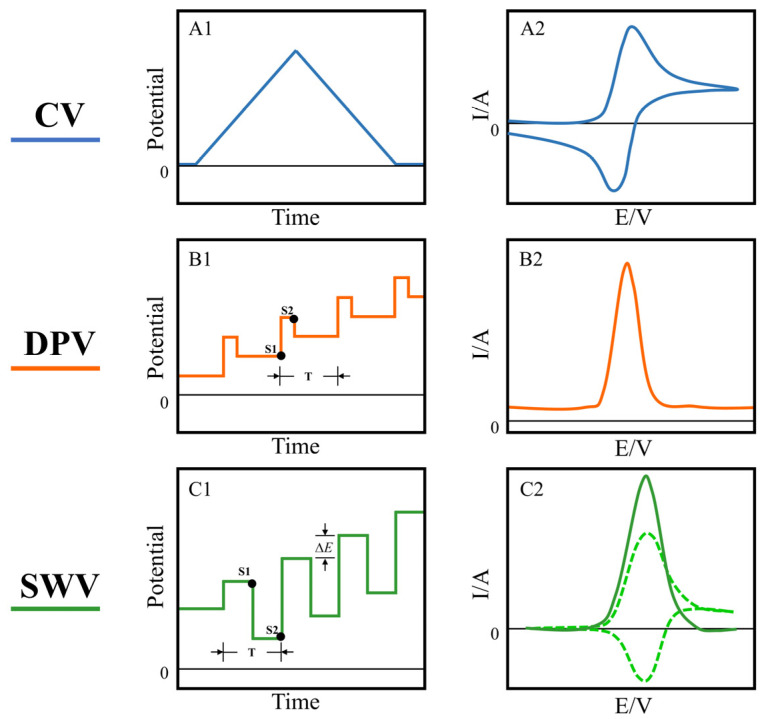
The potential (**A1**) and typical response current (**A2**) of cyclic voltammetry; the potential waveform (**B1**) and voltammogram (**B2**) of differential pulse voltammetry, in the potential waveform, T is the waveform period, and S1 and S2 are the two current sampling points; the typical potential waveform (**C1**) of square wave voltammetry, ∆E is the potential increment, T is the potential period. The response current consists of forward (anodic current) and reverse (cathodic current) components (dashed line in (**C2**)), and their difference results in a net current (solid line in (**C2**)).

Cyclic voltammetry is the most widely used electrochemical technique, which applies a scanning potential of a triangular waveform to a working electrode to detect a generated current (Figure 1A1). Potential cycling at the working electrode drives continuous oxidation and reduction reactions of electroactive species in solution [43]. As the potential applied on the working electrode approaches the equilibrium potential of the species in solution, the response current gradually increases and the oxidized/reduced species on the electrode surface gradually decreases. Until the charge transfer and diffusion motions reach equilibrium, an oxidation/reduction peak appears (Figure 1A2). By analyzing the form of peaks in the voltammogram, information concerning the reaction mechanism, such as the reversibility of reaction, redox potential, reaction rate and concentration of the analyte, can be obtained [44,45]. Pulse-based voltammetry shows an obvious advantage over CV for trace detection. Differential pulse voltammetry superimposes a pulse of fixed amplitude on a step potential. The current is sampled before the potential pulse is applied and again at the end of the pulse (Figure 1B1). Then, the difference can be calculated to get a relatively pure Faradaic current (Figure 1B2). The charging current is represented in the voltammogram as a relatively constant baseline, so background can be better distinguished, enabling a lower detection limit. The good sensitivity of DPV makes it the preferred electrochemical analysis method for trace detection of inorganic and biologically important compounds [46,47,48]. Square wave voltammetry combines the advantages of pulsed and cyclic voltammetry and is considered a more powerful electrochemical technique [49]. Its typical potential waveform is a staircase wave superimposed with a symmetrical square wave. Pulses with equal and symmetrical potential heights are applied to each step plane, and the forward and reverse pulses of each cycle drive the electrodes to undergo electrochemical reactions in both anodic and cathodic directions (Figure 1C1). SWV is able to achieve high scan rates at moderate increments. The strategy of SWV to distinguish the charging current and the Faraday current is to sample the current at the end of each pulse. The two sampling points obtain the forward and reverse currents, and the net current can be obtained by taking the difference between the forward and reverse current (Figure 1C2). SWV and DVP have similar sensitivities, but SWV can achieve a faster detection speed with a shorter potential period. Meanwhile, SWV is suitable for analyzing reversible or quasi-reversible electrode processes. These characteristics make SWV a widely used method in basic research and analysis of biological compounds [50,51,52].

A voltammetric analysis reflects the chemical properties of analytes from different perspectives using functions of potential, current and time. The voltammogram records the current curves of the oxidation and reduction processes of the electroactive species in the analyte and the working electrode at a specific potential. The response current characteristics such as peak value, peak width and peak potential of the voltammetric curve present the electrochemical properties of an analyte. In addition, the response curves also contain redundant information about the solution matrix. The ability of chemometric methods to handle multivariate data offers the possibility to parse more meaningful information from voltammograms.

## 3. Chemometric Tools in Biosensing

Chemometric tools can obtain reasonable analytical results from voltammetric data to improve the performance of voltammetric sensors in biosensing [53,54]. On one hand, the calibration of voltammetric biosensors by simple linear regression presupposes that the desired specificity is present or that the target solution to be detected has been adequately treated. The inhibitory or synergistic effects of interfering substances on the primary analyte must be considered when attempting to detect a single substance in samples with complex matrices. Chemometrics offers a simpler and more flexible approach than spending a lot of effort in improving biosensor selectivity from a sensor design perspective [55,56]. On the other hand, a bioelectronic tongue system consisting of several electrodes with cross-sensitivity is not only capable of measuring a single substance, but can also exhibit a stable differential response for a group of analytes [9,10,57]. In particular, a multi-sensor system using voltammetry expresses rich information about an analytical solution. The richness of this information comes not only from the sufficiently broad cross-sensitivity properties of each sensor, but also from the voltammogram’s complete recording of the electrochemical behavior of the analytes involved in the oxidation/reduction reaction. Chemometric methods provide a critical driving force to process the complex information acquired by multi-analyte sensing systems efficiently [58].

**Figure 2:**
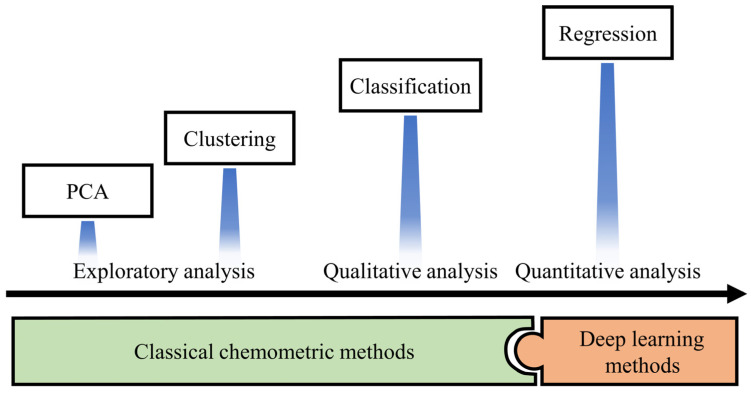
Types of applications of chemometrics. Among them, PCA means Principal Component Analysis.

Chemometrics is defined as a chemical discipline that uses mathematics, statistics and formal logic to provide a vast number of tools for the analysis of voltammetric data [9,57,59]. Although the analysis methods of voltammetric data emerge in an endless stream, they can still be summarized according to the purpose of their analysis (Figure 2).

● Exploratory analysis of the data to obtain the relationship between the data.
● Qualitative analysis of target analytes to deal with the classification and discrimination of samples.
● Quantitative prediction of analytes to achieve the determination of indicators of interest for analytes.

Research papers published in the field of biosensing in recent years have shown a strong interest in deep learning models [60,61,62,63,64]. Compared with widely used classical chemometric methods, the deep learning model shows stronger data transformation ability and wider generality [65,66,67,68]. At the same time, with maturity of machine learning platforms, machine learning models including deep learning can be easily applied to voltammetric data analysis in the field of biosensing.

Validation of method performance and evaluation of effects are also important parts of data processing. Due to the strict requirements in the fields of sensing equipment, experimental process and data measurement, a voltammetric sensing data set often has a small amount of data and uneven samples. In the case of limited samples for analysis, careful consideration should be given to the correctness of method application to establish meaningful correlations between voltammetric biosensor responses and analytical parameters. At the same time, appropriate and reasonable validation strategies were provided for the data processing methods to evaluate the reliability of the models in practice and to avoid inadvertent formation of biased results during data processing and model use.

### 3.1. Classical Qualitative Analysis Methods

Principal Component Analysis (PCA) is one of the most effective and popular analytical tools for data exploration. It reveals the underlying relationships and structure of data in an unsupervised manner, in the absence of prior information about the data [69,70,71]. PCA projects high-dimensional data into a data subspace characterized by principal components (PCs) by determining the maximum variance of the data. Each PC is a linear combination of the original variables, the first PC is the transformation in the direction of maximum variance, and the second PC achieves the second highest variance explanation under the constraint of remaining orthogonal to the first PC. Subsequent PCs complete the interpretation of the remaining information in this manner until a variance cutoff is reached. Therefore, PCA has the ability to make a representation of complex information (score map) in a two-dimensional plane or three-dimensional space, according to the characteristics of the sample. At the same time, the contribution of the original variable to PC can be obtained by the coefficient (loads) of the variable in the linear expression of PC. Therefore, PCA can be used to evaluate the role of each sensor in the electrode array in describing the data relationship. However, this is based on the premise that the information contained in the maximum variance is relevant to the analyte to be analyzed.

**Figure 3:**
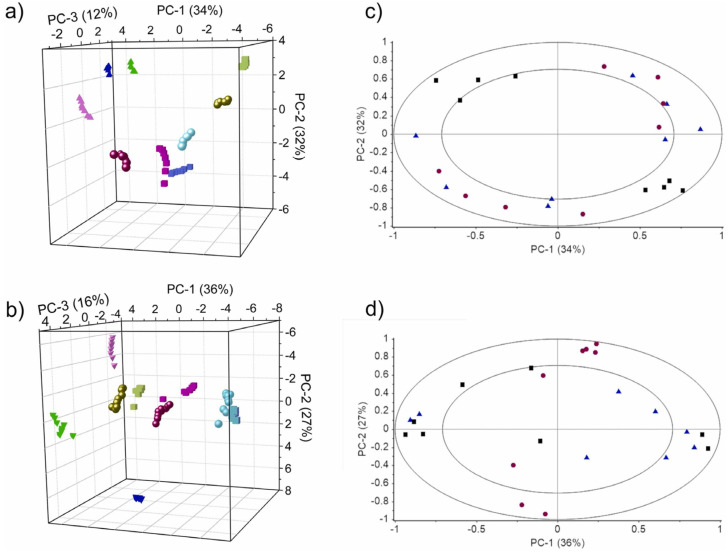
(**a**) Plot of PCA scores for AgNW/bioET and (**b**) AgNP/bioET. (**c**) PCA loading plots of AgNW/bioET and (**d**) AgNP/bioET. Results obtained from milk with different fat content: [skimmed (pink), semi-skimmed (blue) and whole (green)]; or nutritional profiles: classic milk (circles), calcium-enriched milk (triangles), lactose-free milk (square). In the loading plot, the biosensor arrays are represented by β-Gal/AgNWs or β-Gal/AgNPs (black squares), GaOx/AgNWs or GaOx/AgNPs (red circles) and GOx/AgNWs or GOx/AgNPs (blue triangles). Reprinted with permission from Ref. [72]. Copyright 2022 Elsevier.

The score plots and loading plots obtained by PCA are used to show the sample distribution in relation to the study parameters and the contribution of different sensors to the principal components. Coral Salvo-Comino et al. compared the performance of silver nanowires and silver nanoparticles as platforms for immobilizing specific enzymes by scoring and loading plots drawn by PCA (Figure 3) [72]. Different principal components in PCA may contain relevant information about sample parameters; along the PC-3 axis in Figure 3a,b, milk samples are distributed from positive to negative values according to fat content. The loading plot of PCA describes the ability of the electrodes to discriminate against the sample. The results in Figure 3c,d show that the individual values are uniformly distributed in the annular region reflecting the complementary properties between the used biosensors. The score plot and loading plot obtained by PCA can help in further evaluating the performance of silver nanowires as immobilization scaffolds for enzymes in the milk identification task.

PCA enables exploratory studies of sensor performance and analyte parameters. Medina-Plaza et al. immobilized glucose oxidase or tyrosinase on electrodes of six different materials and used PCA to evaluate the ability of an electrode array to identify five grape juices [73]. Through the observation of the PCA score plot, there are clear distances between the five grape juices, and there is a correlation between the distribution positions of the clusters and the content of sugars and phenols in the grape juice. The load plot shows the individual loads of the 12 sensors and provides complementary information. It also shows the redundancy, collinearity and importance of variables in the response matrix. As a complement, clustering in the principal component space was linked to changes in analytes over time. Medina-Plaza et al. developed a nanostructure-based bioelectronic tongue and cyclic voltammetry was used to detect glucose and phenolic content in grape. At the same time, the application of PCA successfully identified the grape harvest year and various stages of growth [74]. The results of PCA demonstrate the ability of the electronic tongue to discriminate grape juice, and the position of the clusters in the score map correlates with the chemical composition. At the same time, the various stages of the grape ripening process are divided into weeks.

PCA can assign samples with similar characteristics to the same cluster according to the inherent properties of the sample data, which exhibits the characteristics of clustering. Strictly speaking, PCA is not a classification method, but it can assist in the judgment of the quality of sample data with class prior information. When there are meaningful clusters in the score plot, it is a reasonable choice to use supervised pattern recognition methods. Class-modeling techniques can supervise the establishment of classification rules to provide qualitative answers to questions according to the needs of the target task. K-Nearby (K-NN), Linear Discriminant Analysis (LDA), Quadratic Discriminant Analysis (QDA) and Partial Least Squares Discriminant Analysis (PLS-DA) are all widely used classical classification methods [59,75,76]. To a certain extent, these methods are simple and effective enough to perform as well or even better than complex methods in certain applications of processing biosensing data [77].

Apetrei et al. used a screen-printed electrode array and cyclic voltammetry based on six electroactive compounds and nanomaterials to monitor the water quality of the Danube for pH, resistivity, turbidity, total dissolved solids, iron content and nitrate ions parameters [78]. An exploratory analysis of the voltammograms by PCA was performed to evaluate the distinguishability of the seven water samples and the contribution of different sensors to the differences in the water samples. At the same time, discriminant factor analysis (DFA) and PLS-DA were used to distinguish the water samples. In addition, ML Rodríguez-Méndez et al. developed two multi-sensing systems based on screen-printed electrodes modified with carbon paste or phthalocyanine to monitor the spoilage of fish meat by analyzing biogenic amines using SWV electrochemical analysis [79]. The score plot of PCA shows the clusters of fish freshness over time, and further, the use of PLS-DA to classify the date of fish degradation according to the sensor array response clearly identifies the various stages of fish degradation. Similar to this work, I.M. Apetrei et al. described the cyclic voltammetry of voltammetric sensor arrays based on bisphthalocyanine- and polypyrrole-modified screen-printed electrodes for amine compounds, including the electrochemical response of ammonia and putrescine [80]. PCA was applied to observe the 4 clusters corresponding to the distribution of days, and then PLS-DA was used to classify the analyzed samples over ten days. The results, validated using the leave-one-out method, show that the sensor arrays developed with these two electrodes for beef freshness monitoring are able to differentiate samples based on their storage time.

It is worth noting that more learning methods for machine classification modeling, such as decision tree (DT) and support vector machine (SVM), have been added to chemometrics, which continue to promote the development of biosensing data analysis. It also provides new strategies for solving quantitative descriptive problems [81,82,83]. Nian Liu et al. have reported an analytical work on the cyclic voltammograms of tea obtained from metal oxides (SnO_2_, ZnO, TiO_2_, Bi_2_O_3_) modified nickel foam electrodes [84]. The principal components with 98% variance obtained by PCA were selected as the modeling data of SVM, and a variety of black tea and green tea were qualitatively classified.

### 3.2. Classical Quantitative Analysis Methods

Quantitative predictions of analyte indicators can be obtained by processing data from voltammetric sensor systems using multivariate calibration methods. Partial least squares (PLS), a classic statistical regression model, provides an effective way for quantitative prediction [85]. Unlike traditional regression models, PLS models are driven by a certain number of latent variables (LVs). Similar to PC in PCA, PLS employs modeling of predictor and response variables to find latent variables that can represent the correlation between the two. However, in the selection of each group of latent variables, the maximization of variance projected on the respective principal components and the maximization of the correlation between the two should be considered at the same time. Starting with the first latent variable, subsequent latent variables are all obtained in the same way from residuals subtracting information from previous variables. Therefore, PLS projects the predictor and response variables, and then completes the regression in this new dimensional space. It is worth noting that the choice of the number of LVs is a more critical option that can be optimized. In general, the number of LVs after crossing the optimal value is proportional to the degree of overfitting of the model.

**Figure 4:**
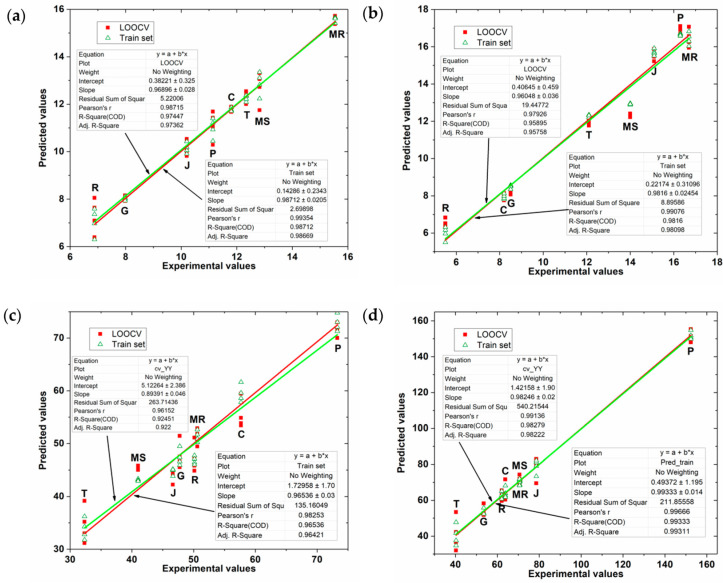
The relationship between the FC and TPC indices of grape seed and grape skin extracts predicted by the PLS model and the experimental values. (**a**) FC index and (**b**) TPC index of grape skin extract; (**c**) FC index and (**d**) TPC index of grape seed extract. The red squares represent the data from the leave-one-out cross-validation set, and the green triangles represent the training set data. Reprinted with permission from Ref. [86]. Copyright 2020, MDPI.

Cristina Garcia-Cabezon et al. used biosensor arrays and PLS to analyze phenolic content in the residues (seeds and peels) of eight grape winemaking species [86]. Figure 4 shows the prediction of FC index (total phenolic content by using the Folin–Ciocalteu method) and TPC index (total polyphenolic content by measuring absorbance at 280 nm) by the PLS model. It can be seen that the PLS model gives good prediction results.

The PLS method has shown high efficiency for high-dimensional variable processing. Clara Pérez-Ràfols et al. used a voltammetric tongue composed of four screen-printed electrodes modified with different materials to detect Cd(II), Pb(II), Tl(I), In(III), Zn(II) and Bi(III) for multivariate analysis of very complex mixtures of metal ions [87]. In order to reduce the large amount of data brought by each sample, they constructed a hierarchical PLS model. The data set was divided into 4 blocks corresponding to the sensor, and PLS was performed separately for each block, and the obtained hidden variables were put into the PLS operation again as new features. Simultaneous quantitative determination of Cd(II), Pb(II), Tl(I) and Bi(III) was achieved in samples in the presence of Zn(II) and In(III) interferences using the proposed hierarchical PLS model. The system provided results comparable to ICP-MS at ppb levels.

PLS has good scalability for different task requirements. Dionisia Ortiz-Aguayo et al. used a voltammetric sensor array composed of three different screen-printed electrodes modified by graphite, cobalt(II) phthalocyanine and palladium. Characteristic voltammograms for heroin, morphine, codeine, caffeine and paracetamol were extracted using SWV [88]. Next, a quantitative model was established to quantify each analyte individually by genetic algorithm and PLS. In order to objectively evaluate the predictive performance of the model in three drug mixtures and mixtures containing two cutting agents, training and testing subset were scientifically established. A full factorial experimental design and a center-complex face-centered experimental design were used to obtain training subsets with good sample distribution, while additional samples in which concentrations were randomly distributed in the experimental domain were prepared to form independent test subsets. In addition, a supplementary test of the permutation test was performed on the data set to prove that the model was not overfitting. An independent test set enables an objective evaluation of the generalization ability of the model’s predictive performance. Ultimately, the results of the model quantifying the mixture of heroin, morphine, codeine, caffeine and paracetamol at the µM level are convincing.

PLS is a very general method that can provide an efficient and stable solution to complex forecasting problems. However, there are a large number of nonlinear factors between predictors and response variables in practical biosensing applications, and classical PLS is weak for modeling data with nonlinear information characteristics. In contrast, artificial neural network (ANN) is a flexible nonlinear model [89]. ANNs are inspired by early models of sensory processing in the brain. Its typical structure is a feedforward network composed of multilayer perceptrons, including an input layer, several hidden layers and a final output layer. The output of each layer of neurons in the network multiplied by the weights of the connections is summed in the next neuron, and then transformed linearly or non-linearly through an activation function. The type of activation function determines whether the model can capture linear or non-linear relationships in the data. In model training, gradient descent is often used to optimize parameters, and the goal of minimization is selected through the loss function, and finally the neural network that can best solve the problem is obtained. It should be noted that, when there are too many parameters for the ANN to learn, the probability of overfitting would become larger. Although this is a common problem with most regression models, the structure of the ANN makes it more prone to over-parameterization. Therefore, the evaluation of the generalization ability of the model is more important.

**Figure 5:**
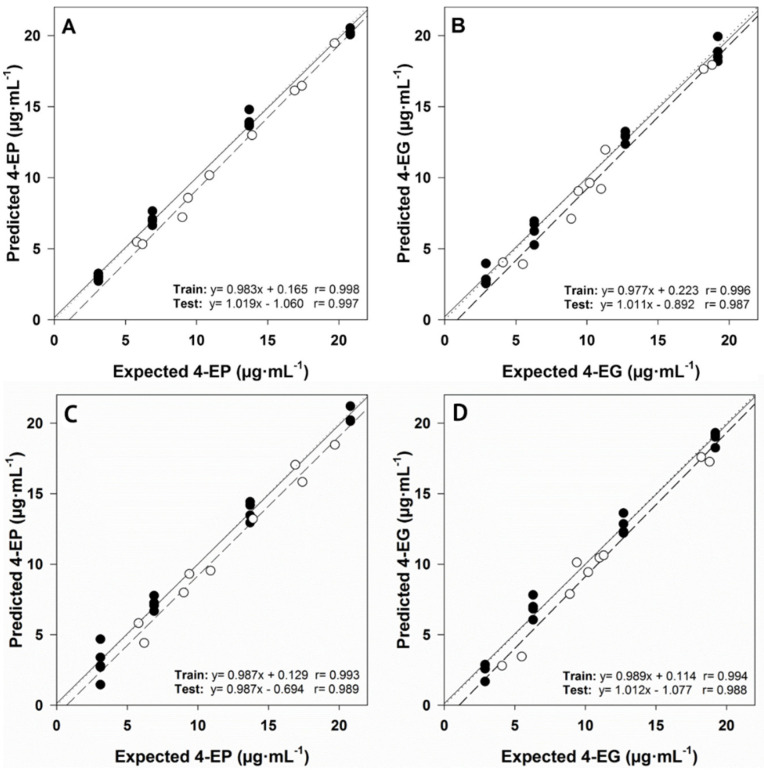
Results obtained using the ANN model: (**A**) 4-EP and (**B**) 4-EG; results obtained using the PLS model: (**C**) fitting of predicted and expected concentrations of 4-EP and (**D**) 4-EG, for training (●, solid line) and test subsets (○, dashed line). Dashed lines correspond to ideal behavior (diagonal). Adapted with permission from Ref. [90]. Copyright 2018 Elsevier.

Figure 5 demonstrates the ability of the ANN model to quantify the binary mixture of 4-ethylphenol (4-EP) and 4-ethylguaiacol (4-EG) [90]. During this work, discrete wavelet transform is used to compress the original data. 144 neurons are composed of input layer, a hidden layer containing 3 neurons is designed, and finally, the output layer is composed of 2 neurons. In the comparison of using ANN and PLS model, it can be seen that ANN shows relatively better performance. The combination of linear and nonlinear operations enables ANN to describe data with nonlinear characteristics, which is the main reason why the ANN model has better predictive ability.

ANN provides help for the analysis of signal dissimilarity and improves the qualitative resolution of complex signals. A more accurate type identification is achieved, and a rough estimate of the concentration is proposed. Zhou et al. demonstrated the simultaneous determination of catechol and quinone in municipal solid waste compost using a tyrosinase-based biosensor array and differential pulse voltammetry [91]. The response currents of 22 potentials in the differential pulse voltammogram were chosen as the input vector, the back-propagation algorithm was selected to optimize the ANN model and the feed-forward back-propagation (BP-ANN) model was constructed. Multiple organic compounds in compost extracts bring diversity and non-linearity. Compared with simple linear regression, BP-ANN provides good analytical capabilities for nonlinear feature relationships. Satisfactory results were obtained in an independent test subset. In another work, M. Asadollahi-Baboli et al. applied square wave voltammetry on screen-printed gold electrodes modified by the formation of cysteine self-assembled monolayers on gold nanoparticles, combined with PCA and Tetracycline and cefixime were determined by ANN in biological fluids [53]. PCA is used for dimensionality reduction of the calibration set, and the dimensionality-reduced data is used as a three-layer ANN network with a sigmoid transfer function. Final validation results in the test set demonstrated the model’s ability to simultaneously determine tetracycline and cefixime concentrations. It also exhibits good performance in an environment with substrates.

### 3.3. Deep Learning Methods

Currently, deep learning shows great potential in many fields. Deep learning models can effectively deal with the complex and high-dimensional data obtained from voltammetric biosensors. They use a multi-layered structure to sequentially extract multi-level features of the source data that reflect the patterns contained in the data. Using these patterns, deep learning models can be well applied to prediction tasks. In the face of different data forms and domain requirements, they have evolved various typical architectures. Convolutional Neural Network (CNN) is one of the most popular deep learning models [92,93]. Similar to the stacked structure of ANN, CNN consists of multiple convolutional layers and ends with a fully connected layer. The input data is convolved with multiple filters in the convolutional layer to obtain feature maps. Filters are learned during training to acquire features about the outcome of the task. At the same time, the operation of convolution brings the advantages of sparse connection and weight sharing. A nonlinear activation function can be used after the convolutional layer to achieve nonlinear mapping. Therefore, the convolutional neural network has the ability to extract features hierarchically and fit the data efficiently. Another classic type of deep network is the Recurrent Neural Network (RNN). The hidden layer of RNN can be regarded as a memory unit, and its output is not only affected by the current input, but also by the historical input, so as to continuously update the internal state. Therefore, RNN is often used to process sequences of arbitrary length, which can represent the influence of historical variables in the sequence on the current input data [94].

**Figure 6:**
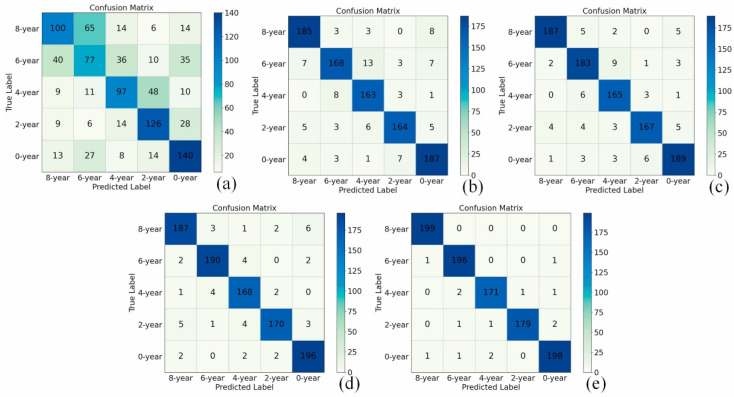
Confusion matrix for Pu-erh tea storage time classification: (**a**) Backpropagation Neural Network (BPNN), (**b**) Extreme Learning Machine (ELM), (**c**) Support Vector Machine (SVM), (**d**) CNN model designed in this paper (**e**) The migrated CNN model. Reprinted with permission from Ref. [95]. Copyright 2021 Elsevier.

Due to its strong data description ability and relatively strong adaptability to new problems, deep learning models gradually enter the field of data processing for voltammetric tongues as a chemometric tool with excellent performance. Figure 6 shows the performance of deep learning models in classification applications [95]. Zheng et al. developed a one-dimensional convolutional neural network to automatically perform feature extraction and classification. At the same time, transfer learning (TL) is also introduced to train the model to enhance the generalization ability of CNN. The research compares the classification performance of the deep learning method used with traditional machine learning methods (back-propagation neural network, support vector machine and extreme learning machine), and the former shows better classification results.

Yuan et al. proposed an automatic feature extraction strategy based on a convolutional neural network in an electronic tongue system for tea classification [96]. Since each sensor provides a large number of current responses for the analyte, it is challenging to extract effective features from the large number of responses that affect the accuracy of pattern recognition. This study built a convolutional neural network (CNN) to learn features automatically. The features in the sensor responses transformed into time-frequency maps by short-time Fourier transform extracted in a supervised training process, which was finally implemented for the classification of five kinds of tea. The research points out that, compared with common classifiers such as SVM, ANN and random forest (RF), the deep model structure that integrates automatic feature extraction and classification into one is more excellent in classification effect.

There are potential pitfalls regarding the use of deep learning models, especially in voltammetric datasets, which often have a small amount of data formed from limited samples. It is necessary to establish effective model verification for deep learning models. A common verification method is to divide the data set into training subsets and verification subsets. The training subset is used in the training process of the model, and the validation subset is used to evaluate the predictive ability of the model. At the same time, the data for the validation subset should remain independent. In small data sets, cross-validation is often used to evaluate model performance. Although this validation method is widely used, it is easy to make mistakes in adjusting the model on the results of the validation set, making researchers overly optimistic about the predictive ability of the model [97]. A suggested validation strategy is to use three subsets of samples: a training subset, a validation subset and a test subset. The optimization of the model based on its performance on the validation subset to find the optimal parameters is especially important for deep learning models. The actual reliability of the final model is estimated by making predictions on the test subset [98]. Since the test subset never participates in the process of model tuning, the evaluation of the generalization ability of the model obtained by this form of evaluation requires affirmation. When the amount of data can only support the division of two data subsets, and the hyperparameters of the model are adjusted during the evaluation process of the validation set, a rational judgment should be maintained on the re-generalization ability of the model.

## 4. Applications of Voltammetric Biosensing in POCT

The combination of chemometric methods and voltammetric biosensors is widely used in food analysis, environmental monitoring, medical and health diagnosis. The small size of the voltammetric biosensor, the simplicity of the voltammetric analysis method and the flexibility of the chemometric method are ideal to obtain the target result from the information obtained by the voltammetric biosensor, for the simple operation and quick analysis of the results in various fields. POCT provides the premise [99]. In particular, devices with excellent computing power and network connectivity, represented by smartphones, provide a good combination for the application of voltammetric sensor systems assisted by chemometric technology for on-site detection and analysis.

**Figure 7:**
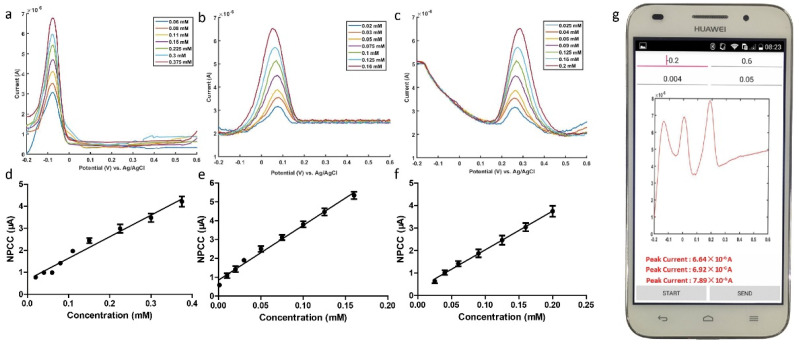
Plot of differential pulse voltammetry measurements of (**a**) ascorbic acid, (**b**) dopamine, and (**c**) uric acid at different concentrations using this system. (**d**–**f**) are dose-dependent curves of ascorbic acid, dopamine and uric acid. (**g**) differential pulse voltammetry detection on the smartphone screen. Reprinted with permission from Ref. [100]. Copyright 2018 Elsevier.

Health-related biomolecular assays can provide reference for disease prevention and diagnosis, and POCT devices play an important role. Daizong Ji et al. designed a smartphone-based voltammetry system for trace detection of ascorbic acid, dopamine, and uric acid using nanomaterial-modified electrodes and differential pulse voltammetry [100]. A smartphone-based integrated voltammetry system includes a sensor, coin-sized detector and smartphone. The detector is used to receive instructions from the mobile phone to realize two electrochemical analysis techniques, cyclic voltammetry and differential pulse voltammetry. The user can control the detection device using the smartphone app, and the voltammogram will be plotted after the detection. Filters for noise processing of current data and acquisition of peaks are also included in the phone (Figure 7). Therefore, the smartphone assumes the functions of controlling the system, processing the data and displaying the data at the same time. The modified electrodes combined with the system in the study can detect not only biomolecular compounds in standard solutions, but also in artificial urine. The results demonstrate that the smartphone-based system can perform simultaneous biomolecular detection, with the potential for instant detection.

**Figure 8:**
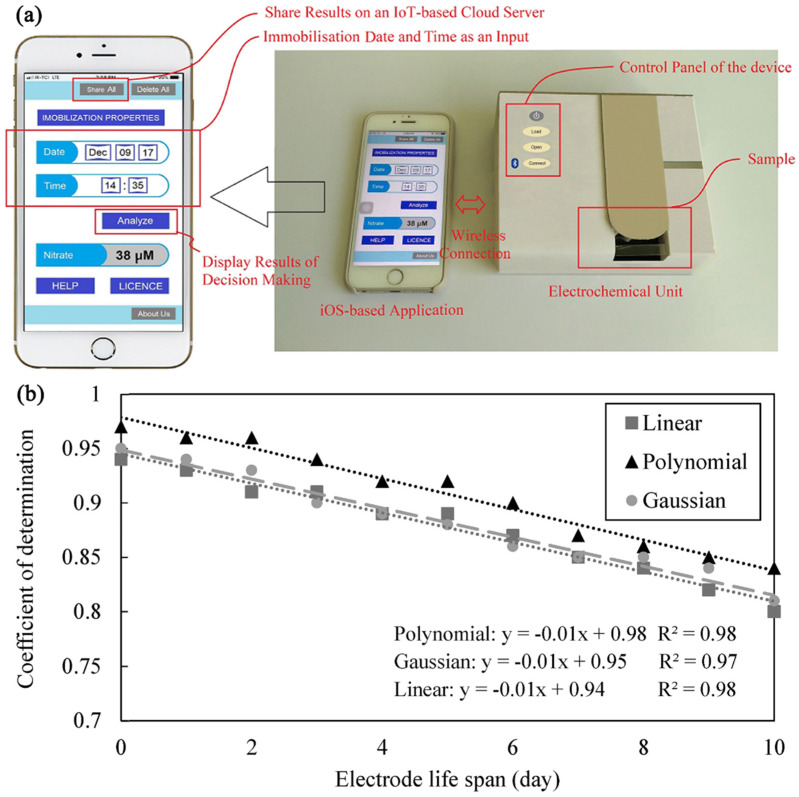
Smartphone-based electrochemical analysis platform and machine learning help with sensor performance. (**a**) Application window of the electrochemical device and smartphone for the determination of nitrate concentration in liquid samples. (**b**) The coefficient of determination (R^2^) of the SVM with different kernel functions in predicting the nitrate concentration of the patterns analyzed at different electrode lifetimes. Adapted with permission from [101]. Copyright 2019 Elsevier.

Smartphones are becoming a versatile platform for developing environmental monitoring POCT systems. Jafar Massah et al. designed a smart portable biosensing system based on enzymes and cyclic voltammetry for the determination of nitrate in liquids [101]. The nitrate concentration was modeled using SVM, taking into account the decrease in enzyme activity over time. The model requires two sets of input features, one is the electrochemical data of the sample and the other is the duration between electrode preparation (enzyme immobilization) and the analysis of nitrate in the sample. Ultimately, the model showed good predictive power (MSE = 0.0018 and R^2^ = 0.92). Meanwhile, the study demonstrates a monitoring system consisting of a portable electrochemical detection device and a decision-making unit based on an iOS platform application and an IoT-based cloud server that shares the results via the Internet (Figure 8). The combination of electrochemical detection equipment and a smartphone with model computing capabilities provides the possibility of instant detection. This work presents a way to deploy trainable machine learning models in smartphones. The community improves the performance of machine learning methods on smartphone applications by sharing databases online. Operators can download the database in any monitoring environment with a network connection, train the machine learning method, and then apply the combination of biosensors, detection device and smartphones equipped with machine learning algorithms for actual monitoring. Benefited by machine learning methods for predicting the nitrate concentrations in samples, this is an instant detection scenario that allows easy access to results without the involvement of a professional chemical analyst.

## 5. Conclusions

Biosensing research has been growing exponentially with the aid of chemometrics. Chemometric tools have shown impressive results both in helping biosensors perform high-resolution detection of multicomponent solutions with matrix effects and in parsing effective information from high-dimensional data generated by bioelectronic tongues. At the same time, the advantages of chemometrics combined with voltammetric-based biosensing have led to the development of point-of-care detection systems that are more accurate, easier to use, and capable of decision making. The current practice of chemometric methods in voltammetric biosensing research provides confidence for more explorations in the future. Here, the status quo is summarized and future directions are as follows:

● Chemometrics is increasingly becoming the dominant driving force of voltammetric-based biosensing research. Chemometrics and machine learning help to better observe and understand the experimental phenomena that result from the interaction of variables under study. It also provides diverse solutions to problems in biosensing while delivering reliable and valuable results.
● Prior research applied deep learning methods to process the responses of voltammetric biosensors or bioelectronic tongues, in stark contrast to the popularity of deep learning in other fields of chemistry. Although the current deep learning models face the problems of small data size and lack of interpretability in the processing of voltammetric data, chemical analysts are still encouraged to learn the core ideas of deep learning. This is not only because the powerful data transformation capabilities of deep learning methods can retrieve meaningful results from complex voltammetric signals, but also because chemical analysts can provide chemical knowledge support for deep learning methods in data analysis of voltammetric biosensing. The contribution of deep learning to other fields of chemistry shows its enormous potential for voltammetric biosensing applications.
● Smartphone-based electrochemical analysis is gradually becoming a reliable solution for POCT in many fields. An easy-to-implement voltammetric method has helped remove the limitations of traditional laboratory assays. Although the implementation of the voltammetric method needs to rely on additional detection equipment, its circuit design and driving method are relatively clear. A large number of portable voltammetric analyzers have been developed. At the same time, the good sensitivity and fast detection speed of voltammetric technology and the great progress made in miniaturization, modularization and cost reduction of biosensing elements (electrodes, detection devices) provide the premise for POCT. Chemometric methods perform a decision-making analysis of the acquired voltammetric data, providing meaningful results for the detection of target analytes. There are reasons to believe that functional devices with mobile computing and multiple connectivity methods represented by smartphones can become the key to the combination of portable electrochemical analysis platforms and chemometric methods. It is expected that an intelligent platform for on-site detection and analysis using voltammetric biosensing systems can be applied in the fields of food industry, environmental monitoring and medical health.

## Figures and Tables

**Table 1:** Biosensing applications using CV, DPV and SWV electrochemical analysis techniques.

Analyte	Electrode	Method	Analytical Parameters	Ref.
LRP gene	Three-dimensional nanoporous gold electrode	SWV, DPV	LOD: 6.0 × 10^−14^ M Linear range: 2.0 × 10^−13^–7.5 × 10^−9^ M	[28]
CYFRA_-21-1_	APTES/nYZR/ITO electrode	DPV	LOD: 7.2 pg/mL Linear range: 0.01–50 ng/mL	[29]
miRNA_-21_	Reduced graphene oxide/gold composite-modified electrode	DPV	LOD: 1.0 pM Linear range: 1 × 10^−14^–1 × 10^−4^ M	[30]
Dopamine; Serotonin; Glucose	GOx-DHP/Gr-AV modified electrode	CV, DPV, SWV	LOD: 0.13 μM Linear range: 30–800 μM LOD: 0.39 μM Linear range: 6.0–100 μM LOD: 0.21 μM Linear range: 1.0–10 μM	[31]
Vitamin D_2_	BSA/Ab-Vd_2_/CD-CH/ITO bioelectrode	DPV	LOD: 1.35 ng/mL Linear range: 10–50 ng/mL	[32]
Promazine	Graphene modified carbon-paste electrode	SWV	LOD: 8.0 nM Linear range: 0.1–8 μM	[33]
Theophylline	CHL-GO/C electrode	SWV	LOD: 4.45 × 10^−9^ M Linear range: 3.0 × 10^−8^–5.0 × 10^−4^ M	[34]
Acetaminophen	Diglycolic acid modified glassy carbon electrode	CV	LOD: 6.7 × 10^−9^ M Linear range: 2.0 × 10^−8^–5.0 × 10^−4^ M	[35]
Osteopontin	RNA aptamer-immobilized gold electrode	CV	LOD: 3.7 nM Linear range: 25–200 nM	[36]
Cardiac troponin I	Au SPE/Au nanodumbbells/Apt	DPV	LOD: 0.08 ng/mL Linear range: 0.05–500 ng/mL	[37]
Troponin I	Au disc/Triangular icicle-like Au	DPV	LOD: 0.0009 ng/mL Linear range: 0.01–5.0 ng/mL	[38]
L-Try	PT-ZnO/glassy carbon	SWV	LOD: 8.5 nM Linear range: 1.0 × 10^−4^–1.0 mM	[39]
Phenol	Tyr-AuNPs/BDD	SWV	LOD: 0.07 μM Linear range: 0.10–11.0 μM	[40]
5-enolpyruvylshikimate-3-phosphate synthase isolated	Dual-functionalized AuNP nanoprobes	DPV	LOD: 0.05 ng/mL Linear range: 0.1–10.0 ng/mL	[41]
Methyl salicylate	AOD-HRP/CNT glassy-carbon electrode	CV	LOD: 0.98 μM Linear range: 0–0.1 mM	[42]

Abbreviations: LRP, lung resistance related protein; APTES, 3-aminopropyltriethyl silane; nYZR, synthesis of yttria-doped zirconia-reduced graphene oxide; ITO, indium tin oxide; miRNA_-21_, microRNA_-21_; GOx, glucose oxidase; DHP, dihexadecyl phosphate; Gr-AV, graphite powder-automotive varnish; BSA bovine serum albumin; Ab-Vd_2_, antibody against the Vitamin D_2_; CD-CH, carbon dots-chitosan; CHL-GO, cholesterol and graphene oxide; C, carbon; AOD-HRP, alcohol oxidase-horseradish peroxidase; CNT, carbon nanotube; L-Try, L-tryptophan; PT, polythiophene; BDD, boron-doped diamond.

## Data Availability

Not applicable.
